# Cerebellopontine angle tumors in infants and children

**DOI:** 10.1007/s00381-015-2747-x

**Published:** 2015-09-09

**Authors:** Tadanori Tomita, Gordan Grahovac

**Affiliations:** Division of Pediatric Neurosurgery, Ann & Robert H. Lurie Children’s Hospital of Chicago, 225 E. Chicago Avenue, Chicago, IL 60611 USA; Feinberg School of Medicine, Northwestern University, Chicago, IL USA

**Keywords:** Brain tumor, Brain stem astrocytoma, Cerebellopontine angle tumor, Cerebellomedullary fissure, Children, Ependymoma

## Abstract

**Objective:**

Cerebellopontine angle (CPA) and cerebellomedullary fissure (CMF) tumors are rare in children and information is scarce in the literature. This retrospective study reports their histological distribution and tumor origin, and describes surgical resections and post-operative outcome based upon the authors’ consecutive personal series.

**Methods:**

Clinical data of infants and children 16 years old or younger of age treated from 2001 to 2012 by a single surgeon was retrospectively reviewed. All had histologically verified CPA/CMF tumors and underwent radical tumor resection through craniotomy except for two children who had a stereotactic biopsy for malignant tumors (glioblastoma and primitive neuroectodermal tumor (PNET)). Tumors’ pathological distributions, tumors’ origin, surgical approaches, and patients’ outcome were reviewed.

**Results:**

There were 44 infants and children with the age at diagnosis ranging from 11 weeks to 16 years; 32 were predominantly in the CPA and/or CMF whereas 12 showed an extension to the fourth ventricle. Pathology showed 14 ependymomas, 12 benign gliomas (11 pilocytic astrocytomas, 1 ganglioglioma), 4 atypical teratoid rhabdoid tumors (ATRTs), 4 epidermoids, 3 primitive neuroectodermal tumors (PNETs), 3 meningiomas, 3 nerve sheath tumors, and 1 glioblastoma. The anatomical site of tumor origin was the lateral recess of the fourth ventricle in 13 patients, the ventral cerebellar hemisphere in 8, the cerebellar peduncle in 7, and the brain stem in 6. Others were from embryonal nest, cranial nerve, or meninges. For 42 tumor resections, 38 were approached through a posterior fossa craniotomy and 4 through a temporal craniotomy and transtentorial approach. At tumor resection, 26 had a gross total or near total resection, 12 subtotal resection, and 4 partial resection. There were no mortalities. The most significant morbidity was ninth and tenth nerve palsy; 15 patients had unilateral vocal cord palsy or dysphagia. Of these, nine were treated with nasogastric (NG) feeding tube, five with a combination of gastrostomy (G-tube) and tracheotomy, and one with G-tube. All had successful removal of NG feeding from 1 month to 2 years (average 6 months). The tracheostomy and G-tube were removed between 4 months and 2 years (average 14 months) in all.

**Conclusion:**

A plethora of tumor types occur in childhood at the CPA/CMF and our review indicated 50 % were benign in histology. High rates of lower cranial nerve morbidity were experienced but their dysfunctions were often recovered or compensated in 2 years. However, one should be cognizant of these complications and conduct resection with appropriate surgical approach, intraoperative monitoring, and surgical microscope.

## Introduction

The majority of posterior fossa tumors in children develop in the midline including the fourth ventricle, cerebellar vermis and brain stem, or in the cerebellar hemispheres. However, the cerebellopontine angle (CPA) is a rare site and less than 10 % of posterior fossa tumors in childhood were located in CPA [1].

The CPA is a subarachnoid space located in the ventral surface of the brainstem and medial cerebellar hemisphere, laterally bordered by the superior and inferior limbs of the cerebellopontine fissure (CPF). The CPF is formed by the ventrolateral wings of the quadrangular lobule (superiorly) and simple lobule (inferiorly) folding around the middle cerebellar peduncle. The superior and inferior limbs of the CPF meet at the lateral corner of the CPA, lateral to the flocculus, and extend laterally to the horizontal fissure of the cerebellum. Inferiorly, the inferior limb of the CPF extends to the cerebellomedullary fissure (CMF), which is bordered by the medulla oblongata and the medial wall of the biventer lobule, forming the lateral cerebellomedullary cistern. Medially at the CPA, the lateral recess of the fourth ventricle opens to the CPA through the foramen of Luschka. The inferior cerebellar peduncle forms the ventral and rostral wall of the lateral recess. In the CPA and CMF, where respective cerebrospinal fluid (CSF) cisterns are present, multiple cranial nerves from the 5th to 12th nerve are present. At the foramen of Luschka, fourth ventricular choroid plexus protrudes into the CPA cistern. Also present are the arterial branches of vertebrobasilar system including posterior inferior cerebellar and anterior inferior cerebellar arteries.

A plethora of tumors can occur in the CPA and CMF, which derive from neuroglial tissues, cranial nerve sheath, meninges, and embryonic remnants [1]. Thus, tumors of this location originate from the structures normally present in this location or embryonic remnants. In the adult, common tumors at the CPA are vestibular schwannoma, meningioma, and epidermoid. According to Bonneville et al., vestibular schwannoma account for 70–80 % of all CPA lesions, 10–15 % meningiomas, and 5 % epidermoid in adults [2]. However, these tumors are rare in pediatric population, and only a limited number of publications are available in the literature during past decade [1, 3-5]. Pediatric CPA tumors are often secondary to an exophytic extension of intrinsic tumors originating from the adjacent brain stem, cerebellar peduncle, and cerebellum or a direct extension from the fourth ventricle and lateral recess, thus the histological distributions differ from the adult counterpart. Sensitive neural and vascular structures in this location often hinder safe aggressive resections. These tumors tend to be large, not necessarily confined to the CPA or CMF, but rather occupy both spaces, and further extend to the neighboring structures.

In this communication, the authors retrospectively reviewed a series of pediatric CPA and CMF tumors focusing on the histological distribution, their tumor origin, surgical approach and techniques, and their outcomes.

## Material and methods

This series is composed of the senior author’s (TT) personal pediatric surgical cases of primary tumors located in the CPA and/or CMF which were diagnosed and treated between 2001 and 2012. All patients had their tumor histology verified. Excluded are those with intrinsic expansion of the brain stem or cerebellum that is confined by the pia. Among tumors that affect both the fourth ventricle and the CPA/CMF, only those with the cisternal portion larger than the fourth ventricle portion are included. Vestibular schwannomas confined within the internal auditory canal(s) are excluded. Primary bony tumors arising from the skull base are also excluded.

## Case summary

### Presentation

There were 44 infants and children in this series. There were 22 females and 22 males. The age at diagnosis ranged from 11 weeks to 16 years. All were evaluated with CT and MRI preoperatively.

Of locations of these tumors, 32 were predominantly in the CPA and/or CMF whereas 12 showed a dumbbell-shaped extension into the fourth ventricle. Pathology of all tumors was verified: 14 ependymomas, 12 gliomas (11 pilocytic astrocytoma, 1 ganglioglioma), 4 atypical teratoid rhabdoid tumors (ATRTs), 4 epidermoids, 3 primitive neuroectodermal tumors (PNETs), 3 meningiomas, 3 nerve sheath tumors, and 1 glioblastoma. The presumed origin of the tumor correlating with the pathology is shown in Table 1. The most common anatomical site of tumor origin was the lateral recess of the fourth ventricle observed in 13 cases, followed by the ventral cerebellar hemisphere in 8, cerebellar peduncle in 7, and the brain stem in 6 cases. Four epidermoids were considered to arise from embryonal nests, and the three meningiomas and three nerve sheath tumors developed from cranial nerves and meninges, respectively.

**Table 1:** Origin and pathology of CPA tumors

		Pathology						
Origin		Ependymoma (14)	JPA (12)	ATRT (4)	Epidermoid (4)	PNET/GBM (4)	Meningioma (3)	Schwannoma (3)
Lateral recess	(13)	13						
Cerebellum	(8)	1	1	4		2		
Cerebellar peduncle								
Restiform body	(6)		6					
Brachium pontis	(1)		1					
Brainstem								
Pons	(4)		2			2		
Medulla oblongata	(2)		2					
Embryonal remnant	(4)				4			
Meninges	(3)						3	
Cranial nerve	(3)							3

At diagnosis, 23 patients were noted to have hydrocephalus. The most common presentation was headaches or irritability/lethargy. Gait disturbances among the children and developmental arrest or regression among infants were common among hydrocephalic patients. Of 44 patients, 15 were found to have cranial nerve dysfunctions preoperatively; hearing loss in 4, dysphagia/dysphonia in 5, facial weakness in 2, facial pain in 2, and sleep apnea and shoulder droop in 1 patient each.

### Surgery

**Fig. 1:**
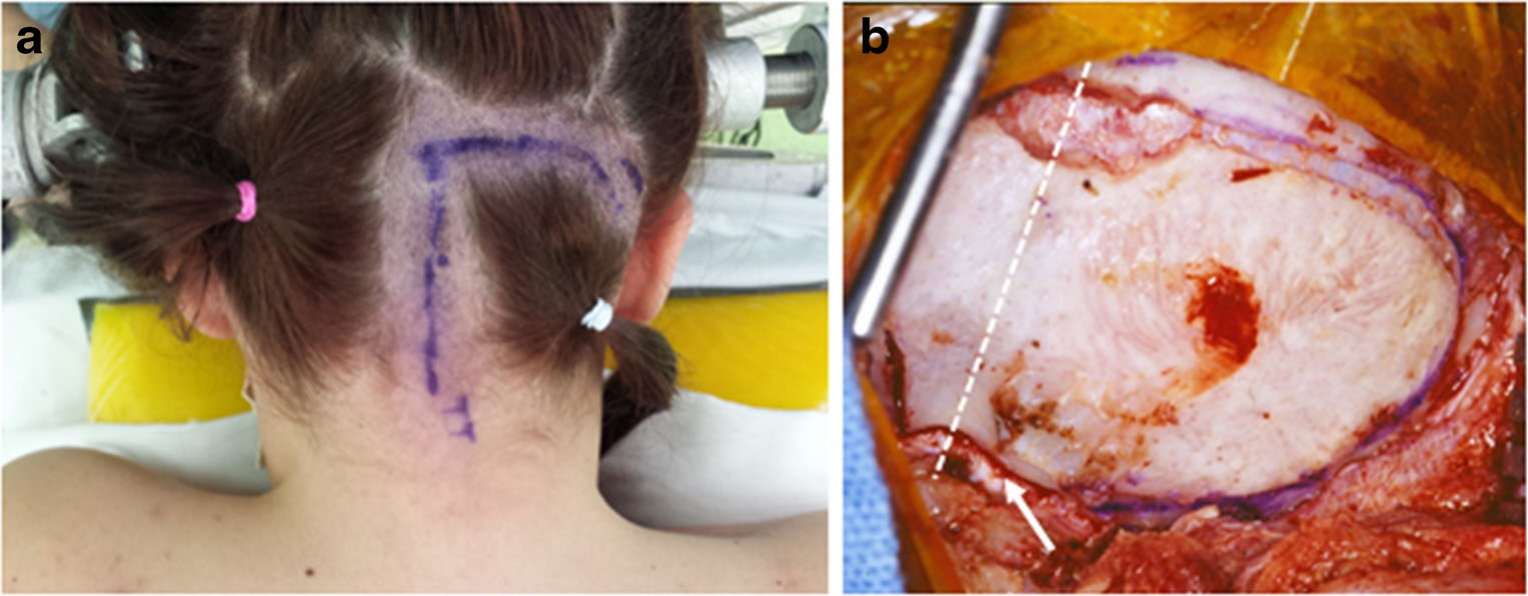
Posterior fossa craniotomy for CPA tumors. **a** A hockey stick incision with a midline incision from C2 spinous process level with an upper lateral incision extending toward the base of mastoid process. **b** A surgical photograph showing a posterior fossa craniotomy of bilateral occipital bone with the ipsilateral side wider, extending to just medial to the sigmoid sinus. A *dashed line* indicates the sagittal midline. Note the craniotomy crosses the foramen magnum (*arrow*)

Of 23 cases with pre-operative hydrocephalus, 3 had a shunt placed elsewhere and all others had external ventricular drainage at the time of craniotomy for tumor resection. All patients had surgical resection through 42 craniotomies and 2 stereotactic biopsies (PNET and glioblastoma). Of 42 craniotomies, 38 patients had a posterior fossa (PF) craniotomy and 4 patients had temporal craniotomy with subtemporal transtentorial approach to the CPA. All were resected with surgical microscope and neurophysiological monitoring. For posterior fossa craniotomy among 38 cases, 36 patients were in the prone position, with 26 patients receiving a “hockey-stick” incision (Fig. 1) with a combination of midline and lateral craniotomy, and 10 patients with a midline incision and a midline craniotomy. The remaining two patients were placed in the supine position with the head turned to the ipsilateral direction and approached through an ipsilateral retro-sigmoid craniotomy. Of 42 patients with intent of radical tumor resection, 26 had a gross total resection, 12 subtotal resection, and 4 partial resection.

### Surgical complications

There were no deaths related to surgical procedures in our series. However, surgical morbidity rates were high: 17 patients had unilateral vocal cord palsy or dysphagia; 2 were noted preoperatively. Of these, postoperatively, 10 were treated with nasogastric (NG) feeding tube, 5 with a combination of gastrostomy (G-tube) and tracheotomy, and 1 with G-tube. All had successful removal of NG feeding from 1 month to 2 years (average 6 months). The tracheostomy and G-tube were removed between 4 months and 2 years (average 14 months) in all. Two had fifth nerve and one had sixth nerve dysfunctions. Two patients with lateral medulla oblongata tumor had a trigeminal trophic syndrome, which occurred 6–15 months postoperatively. Dense hemiparesis occurred in two patients (one persisted) and increased cerebellar ataxia occurred in two others.

### Outcome

**Fig. 2:**
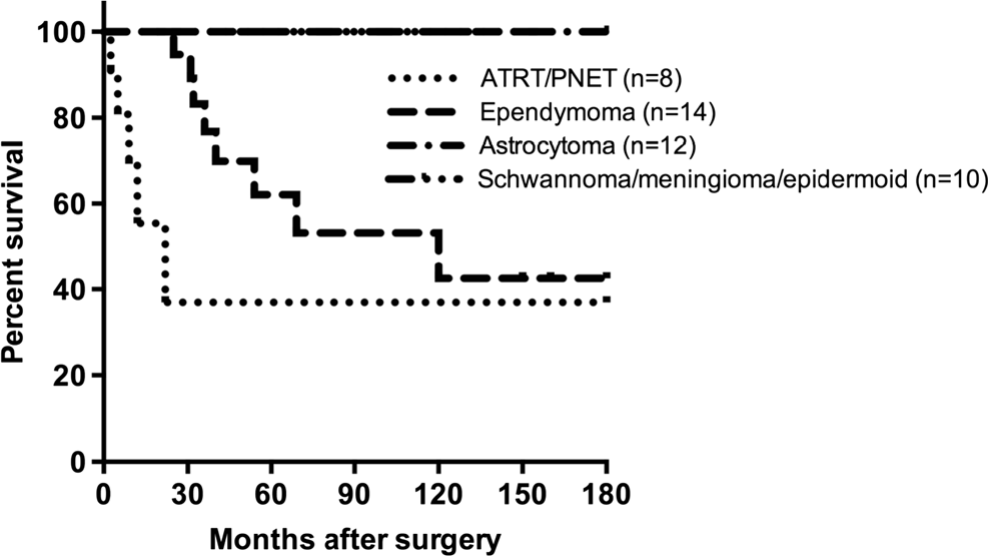
Kaplan-Meier plot of overall survival of patients with pediatric CPA tumors. Both groups of the patients with benign astrocytoma, and those with schwannoma, meningioma, and epidermoid are all alive whereas overall survival rate of those with ependymoma and ATRT/PNET/glioblastoma was 42.8 and 37.5 %, respectively

During the follow-up period up to 15 years, 13 patients were known to be dead. They were 3 of 4 ATRTs, 8 out of 12 ependymomas, and 1 each of PNET and glioblastoma. Both groups of the patients with benign astrocytoma and those with schwannoma, meningioma, and epidermoid are all alive whereas overall survival rate of those with ependymoma and ATRT/PNET/glioblastoma was 42.8 and 37.5 %, respectively (Fig. 2). Death occurred in three ATRT patients between 5 and 9 months after surgery in spite of chemotherapy. One patient with ATRT, who was treated with chemotherapy and radiation therapy (RT), has been disease free over 9 years. This case was reported elsewhere [3]. Eight of 14 ependymoma patients had died due to recurrence in spite of RT. The rest, four patients with and two patients without adjuvant therapy, remain recurrence free after gross total resection for periods of time ranging from 2.5 to 13 years. Of 12 benign glioma cases, one patient received chemotherapy after partial resection and the others were observed without therapy after resection. Two had recurrence; one treated with RT and another with chemotherapy following reoperation.

### Illustrative cases

**Fig. 3:**
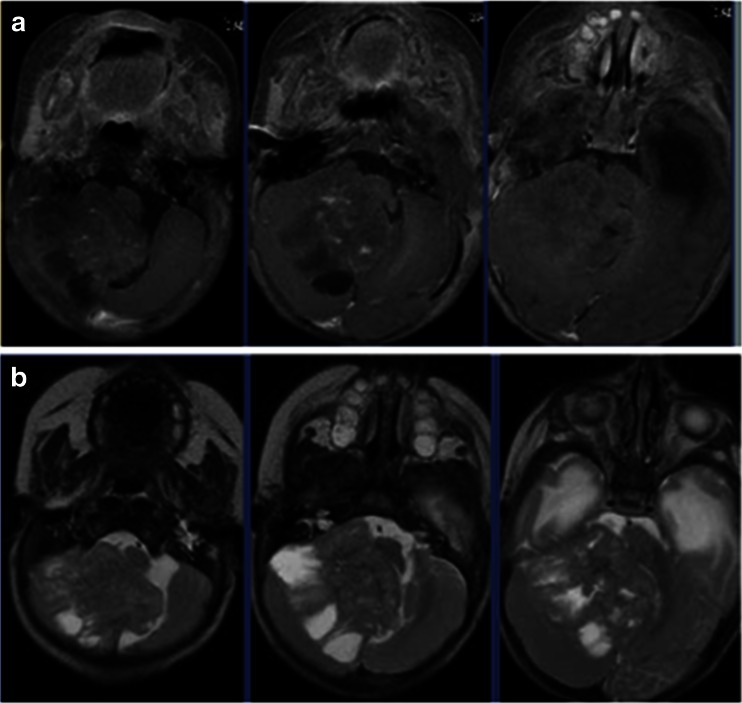
Case 1. Axial MR image (**a** T1-weighted post contrast, **b** T2-weighted) of a 3-month-old infant showing a large poorly enhancing, inhomogeneous right cerebellar ATRT extending into the CPA

**Fig. 4:**
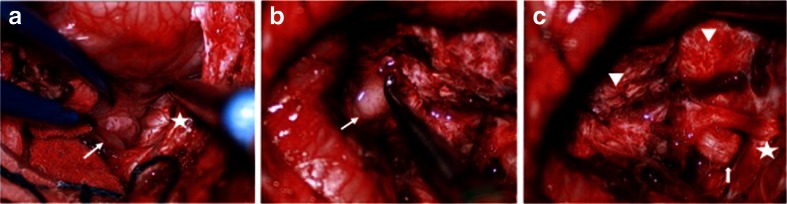
Surgical photograph of case 1. **a** Following the major bulk of tumor from the cerebellar hemisphere, the cerebellomedullary fissure is exposed and the tumor (*arrow*) is separated away from the 9th and 10th nerves and the jugular foramen is exposed (*star*). **b** The fourth ventricle is displaced but not involved. **c** Following resection of CPA/CMF ATRT, the internal auditory meatus (*arrow*) and the jugular foramen (*star*) and respective cranial nerves are shown. Note the cerebellar white matter after tumor resection (*arrow head*)

**Fig. 5:**
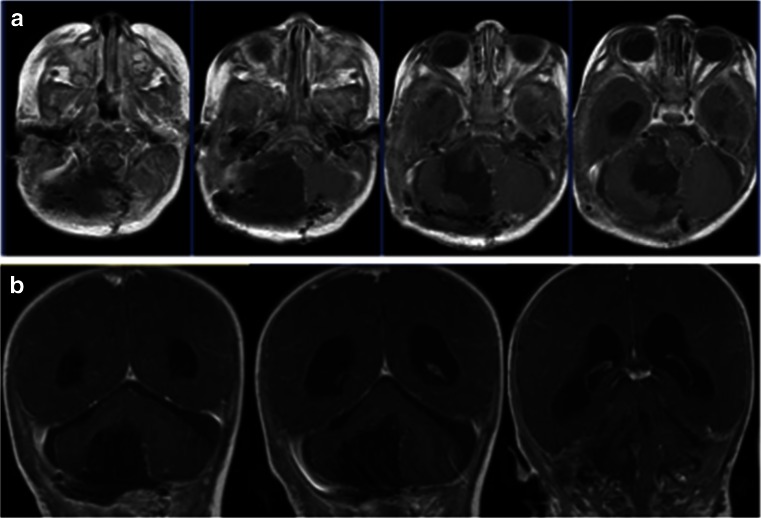
Case 1. Post-operative T1-weighted MRI after contrast infusion (**a** axial, **b** coronal) showing a gross total resection of ATRT
- Case 2. This 12-month-old boy presented with increasing head circumference together with psychomotor developmental arrest for several months. He was also diagnosed to have “arthrogryposis” shortly after the birth which caused a delay in his developmental milestones. MRI showed a marked hydrocephalus due to very large poorly enhancing homogenous tumor extending from the left CPA to the fourth ventricle and cisterna magna (Fig. 6). Through a posterior fossa craniotomy, the tumor was found in the subarachnoid space in the cisterna magna and upper cervical cord. After debulking the tumor and lifting it, the spinal accessory nerve was found in the CMF subarachnoid space and the jugular foramen. Also, the PICA of the left side was traced and the vertebral artery was found. The vertebral artery was surrounded by tumor but dissected free under microscope. The 9th and 10th nerves were at the CMF wrapped around by the tumor, which were also separated and their anatomical continuity was preserved. At the CPA, the tumor was extending to the tentorial surface but seventh and eighth nerve complex was preserved at resection. The tumor further extended into the lateral recess and lateral fourth ventricle space, but was removed. The tumor appeared to have an origin from the cerebellar hemisphere. A gross total resection was attained except for a small portion at the 9th and 10th nerve entry zone to the medulla (Fig. 7). Postoperatively, he developed bilateral vocal cord palsy noted and received tracheotomy and G-tube placement. The pathology showed a tanycytic ependymoma. Postoperatively, he received no adjuvant therapy. A follow-up laryngoscopy a year later showed normal right but reduced left vocal cord mobility. However, 2 years later, the laryngoscopy examination showed normal vocal cord function on both side, and his speech and swallowing functions returned normal. He had the G-tube removed 18 months post-surgery and tracheotomy was decannulated 2 years after surgery. MR done 3 years later showed no signs of tumor recurrence (Fig. 8)

- Case 1. This 3-month-old baby girl presented with frequent emesis and subsequently developed poor head control and prominent scalp veins. Head CT and MR disclosed hydrocephalus and a large inhomogeneous non-enhancing tumor in the right cerebellar hemisphere extending to the right CPA (Fig. 3). She had a posterior fossa craniotomy and the tumor was noted in the right cerebellar hemisphere extending to the right CMF and CPA (Fig. 4a). The fourth ventricle was displaced contralaterally but not involved (Fig. 4b). The 9th, 10th and 11th nerves were engulfed by the tumor at brainstem entry zone but their anatomical continuities were preserved (Fig. 4c). The displaced seventh and eighth nerves were protected and a gross total resection of the tumor was attained on post-operative MR (Fig. 5). Pathology report showed ATRT. Postoperatively, she showed stridor due to right vocal cord paresis and swelling. Also, she had a mild right facial weakness and left-sided esotropia, both of which resolved spontaneously. Speech evaluation indicated that she had moderate-severe oral-pharyngeal dysphagia. She was fed through NG feeding tube. She then received high-dose chemotherapy with stem-cell rescue. She was able to take oral feeding; however, 6 months postoperatively, she passed away due to septic complications of intense chemotherapy. No tumor recurrence was noted.

**Fig. 6:**
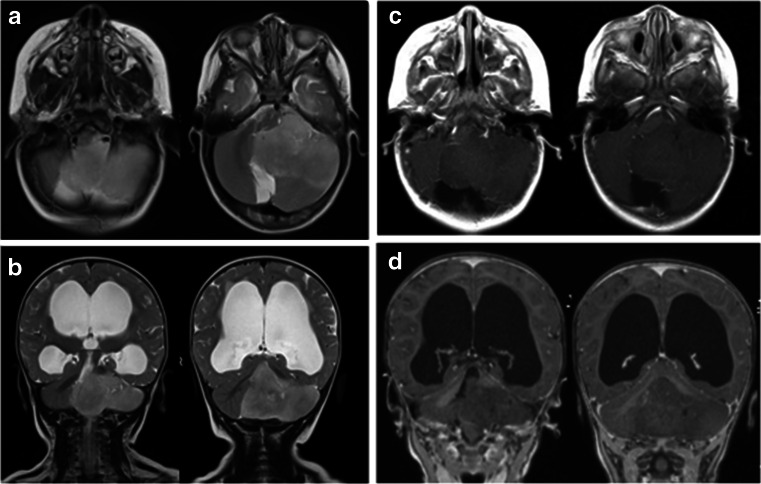
Case 2. Pre-operative MRI (**a** T2-weighted axial view, **b** T2-weighted coronal view, **c** post-contrast T1- weighted axial view, **d** post-contrast T1-weighted coronal view) showing a large CPA tanycytic ependymoma and marked hydrocephalus

**Fig. 7:**
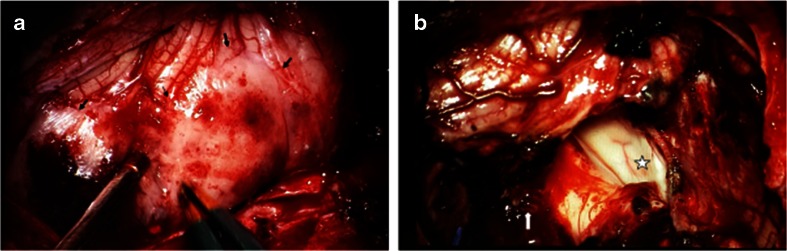
**a** Surgical photograph of case 2 showing a large tumor (*arrows*) extending into the cisterna magna. **b** Post-resection showing a enlarged lateral recess to the fourth ventricle (*star*). Ninth and 10th nerves are shown (*arrow*) and there is a small residual tumor left surrounding the rootlets of these nerves

**Fig. 8:**
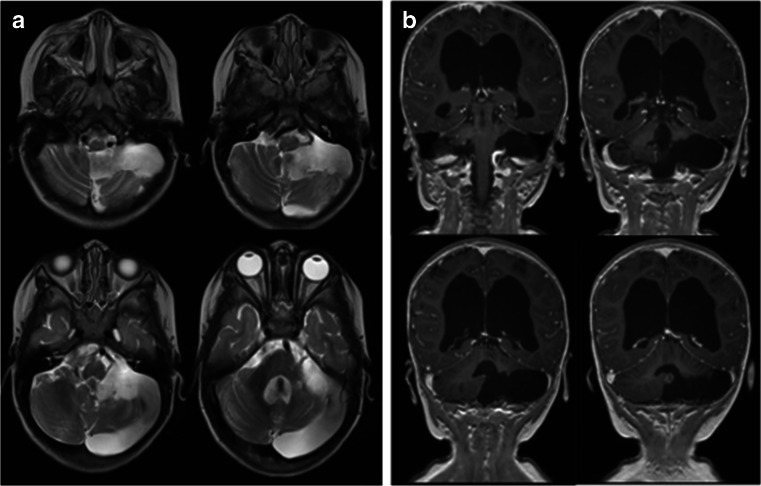
Case 2. Three years post-operative MRI (**a** T2-weighted axial view, **b**, T1-weight coronal image after contrast) showing no signs of residual or recurrent tumor
- Case 3. This is a 4-year-old girl who presented with intermittent headaches with fever since a year ago. Due to increasing episodes of “chemical meningitis,” she had head CT and MR, she had CT and subsequent MRI which showed a large non-enhancing mass in the midline ventral to the brain stem at the pontomedullary junction (Fig. 9). Pre-operative neurological examination was normal, however. Pre-operative laryngoscopy showed normal vocal cord movements. She had a PF craniotomy with the bone flap primarily on the left occipital bone crossing the midline and crossing the foramen magnum (Fig. 10a). The CMF and CPA were entered by lifting the cerebellar tonsil and hemisphere of the left side (Fig. 10b). Medially to the 9th and 10th nerves and also to the 7th and 8th nerve complex was the cloudy capsule of an epidermoid (Fig. 10c). Clearly, debris from the epidermoid contents was noted floating in the CSF space. The main approach was through the space below the 10th nerve and another above the 9th nerve. The lesion was removed from the space between the basilar artery and the ventral surface of the brain stem (Fig. 10d). Postoperatively, she developed a left vocal cord palsy, needing G-tube. She started to take oral feeding by 3 months and G-tube was removed 6 months postoperatively. At the last examination 4 years following the surgery, she had normal neurological function and follow-up laryngoscopy showed normal vocal cord function. No signs of epidermoid recurrence were noted on follow-up MR (Fig. 11)

**Fig. 9:**
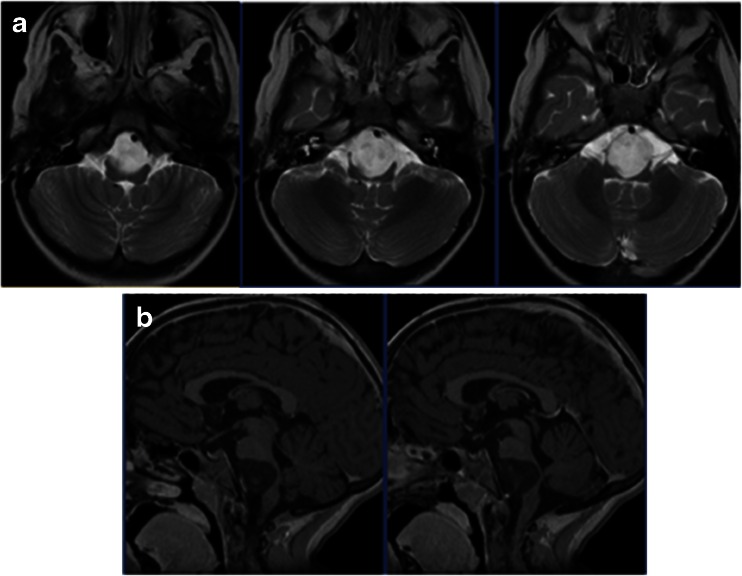
Case 3. Four-year-old girl with previous history of chemical meningitis. MR images (**a** T2-weighted axial view, **b** T1-weight sagittal image after contrast infusion) showing a large mass ventral to the ponto-medullary junction. Note a severe brain stem compression by the non-enhancing tumor between the brain stem and the basilar artery

**Fig. 10:**
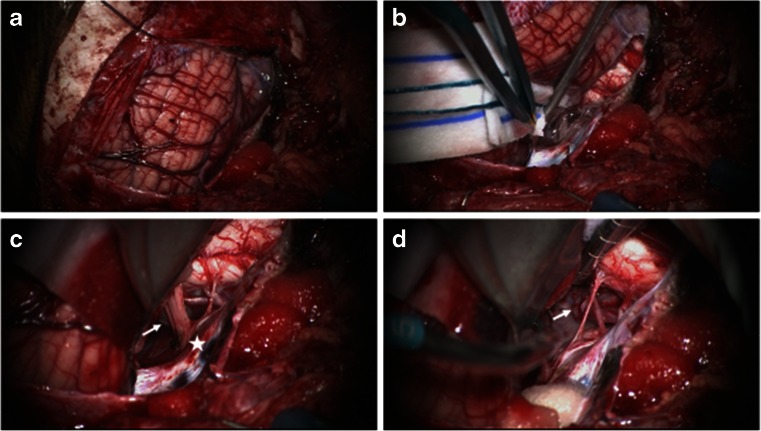
Surgical photographs of case 3. **a** Through a hockey stick incision and predominantly left posterior fossa craniotomy and dural opening, the left cerebellar hemisphere and tonsil are exposed. **b** By lifting the left cerebellum and tonsil, the left jugular foramen and cranial nerves are identified. **c** The jugular foramen (*star*) and the 9–10th nerves are identified in the CMF. The epidermoid capsule is noted ventral to the brain stem surrounded by lower cranial nerves. **d** Following the epidermoid resection, the vertebrobasilar artery (*arrow*) is noted

**Fig. 11:**
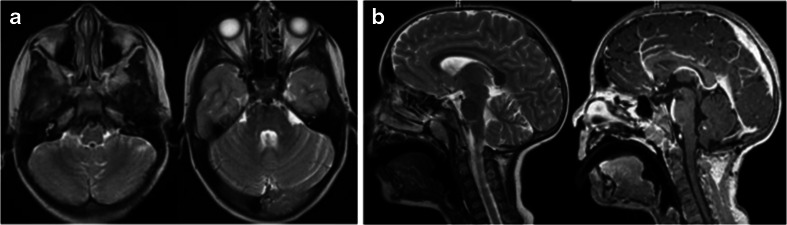
Case 3. Post-operative MRI (**a** T2-weight axial image, **b** T1-weight sagittal image after contrast) showing no evidence of residual or recurrent epidermoid
- Case 4. This is a 5-year-old boy who presented with increasing lethargy after a minor fall a week ago. On examination, he was noted to have speech dysarthria with decreased gag reflex on the left side. He has nystagmus at lateral and upward gaze, and a mild left-sided dysmetria. Head CT and subsequent MR showed a large mass in the left CMF without hydrocephalus (Fig. 12). Through a hockey stick incision and posterior fossa craniotomy and C1 arch resection, a large exophytic tumor was exposed in the cisterna magna and upper cervical subarachnoid space (Fig. 13). The tumor appeared to be originating from the posterolateral medulla oblongata dorsally to the root entrance of 9th and 10th nerves. It extended medially to the lateral wall of the fourth ventricle, rostrally into the cerebellar parenchyma and laterally and anteriorly into the CPA. A gross total resection was attained sparing all visible cranial nerves of 7th to 11th. The pathology showed a juvenile pilocytic astrocytoma (JPA) and it appeared to be originating from the inferior cerebellar peduncle. Postoperatively, he had dysphagia necessitating NG feeding. He had supplemental NG feeding for 2 years which was then stopped. He had to turn his head to the left side to assist swallowing for big meals. He did not receive any adjuvant therapy for the tumor. Presently, 14 years after the resection, he attends college and his speech and swallowing were normal. The only neurological findings were a slight deviation of uvula and soft palate, minimal dysmetria of the left side, and left neurosensory hearing loss. The latest MRI showed no evidence of tumor (Fig. 14)

**Fig. 12:**
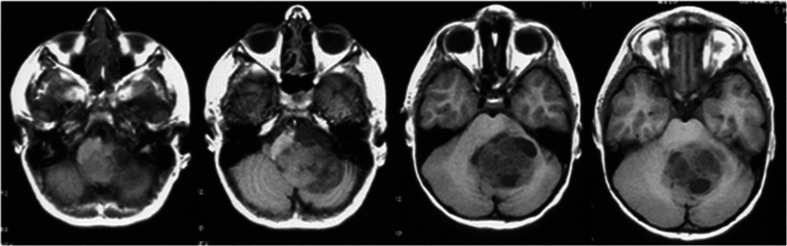
Case 4. Pre-operative T1-weight axial MRI showing a large heterogeneous tumor in the left CMF and CPA displacing the fourth ventricle

**Fig. 13:**
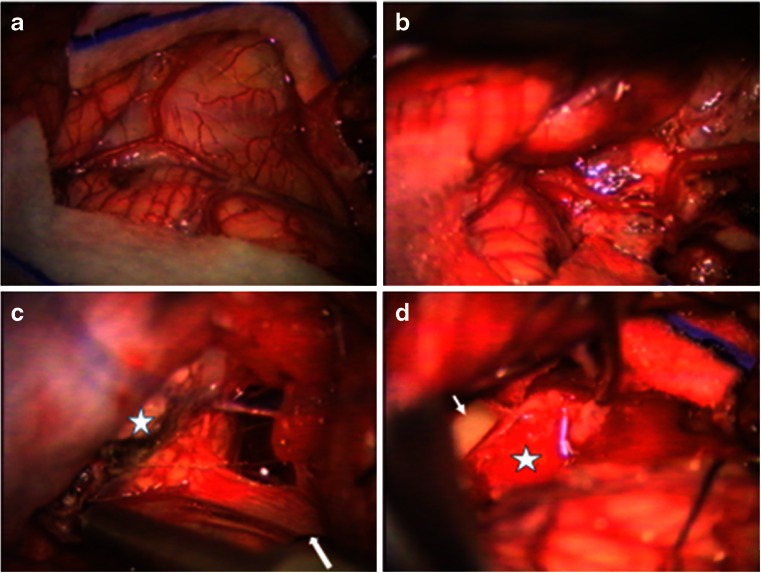
Surgical photograph of case 4. **a** Large exophytic JPA extending into the cisterna magna. **b** Following the resection of the exophytic tumor, the dorsal spinal cord is uncovered together with left posterior inferior cerebellar artery. **c**, The tumor further extends into the lateral dorsal aspect of the medulla oblongata, which was removed. Note the inspection of the lateral medulla oblongata by turning it inward following the tumor resection. Note the 9th and 10th nerves are shown entering the jugular foramen (*arrow*) and medially there is a tumor resected cavity (*star*). **d** The JPA appeared to originate from the inferior cerebellar peduncle and a gross total resection was attained. Note the open fourth ventricle (*arrow*) and the tumor resected cavity wall at the dorsal medulla oblongata and upper cervical cord (*star*)

**Fig. 14:**
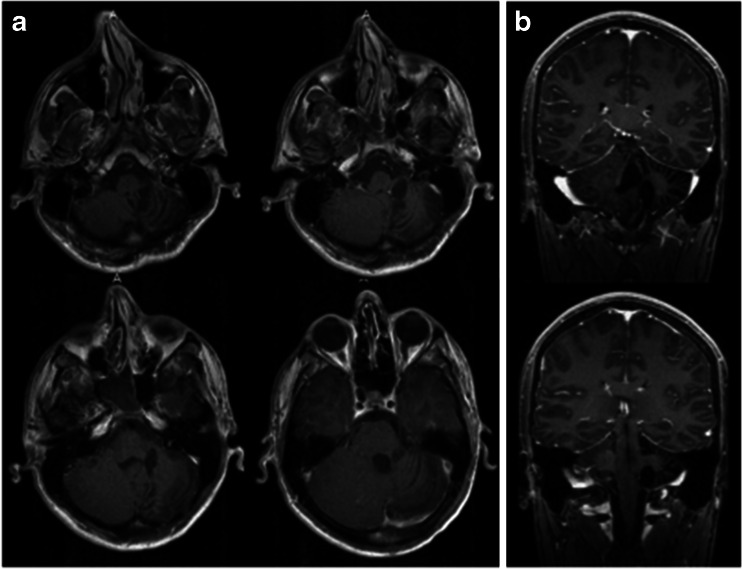
Case 4. Post-operative T1-weighted postcontrast MRI (**a** axial, **b** coronal). There is no evidence of disease

## Discussion

### Tumor pathology and origin

For children with CPA and CMS tumors, advanced neuroimaging provides the location and extension of the tumor. Extrinsic tumors in the cistern, particularly those of nerve sheath origin of the cranial nerves and meninges are considered to be pure CPA/CMF tumors. Similarly considered are the tumors originating from embryonal remnants, such as epidermoid and neurenteric cyst. However, tumors primarily arising from brain stem, cerebellar peduncle, and anterior cerebellar hemisphere can predominantly take an exophytic extension to the cerebellopontine and/or cerebellomedullary cistern. Theses tumors with exophytic extension may mimic extrinsic tumors. Matson described nine cases of gliomas (four astrocytomas, four mixed tumors, and one ependymoma) in the CPA with no obvious point of origin from the cerebellum or brain stem [4]. However, in our series, all tumors had an identifiable origin. Pre-operative imaging may not distinguish the point of origin, but this can be determined based upon the histological type, intraoperative observation, and post-operative imaging [5]. In our series, 34 were exophytic intrinsic tumors, and 10 were extrinsic, such as nerve sheath tumor, meningioma, and epidermoid.

Thirty-two of our patients were predominantly in the CPA and/or CMF cisterns whereas 12 showed a dumbbell-shaped extension between the fourth ventricle and the CPA. Tumors, particularly those predominantly in the CMF, may look like they are growing into the fourth ventricle cavity, but they may be displacing the fourth ventricle contralaterally without internal invasion. Tsai et al. classified 29 pediatric CPA tumors into three categories: those exclusively confined in the CPA (8 patients), those with tumors predominantly within CPA (16 patients), and those arising from the vicinity and growing into CPA (5 patients) [6].

In the CPA and CMF, a plethora of tumors occurs, but they are rare during childhood, only representing 1 to 3 % of pediatric brain tumors. According to Phi et al., the CPA and CMF location consists of only 10 % of posterior fossa tumors [1]. Case studies vary regarding the predominant tumor type, ranging from benign tumors [7] to more malignant phenotypes [1]. In adults, the majority of tumors arising in the CPA are benign extra-axial tumors such as vestibular schwannomas, meningiomas, and epidermoid cyst. However, vestibular schwannoma and meningioma are rare in childhood, except for patients with neurofibromatosis type 2 (NF2). According to Holman et al., schwannomas are the most common pediatric tumor type: 57 of 87 CPA and internal auditory tumors (IAC), followed by meningiomas (5/87) and epidermoid cysts (4/87) [8]. In their series, there were only four exophytic gliomas (two each of pilocytic astrocytoma and gangliogliomas) and six various malignant tumors. They observed a high incidence (61 %) of neurofibromatosis 2 (NF 2) among the vestibular schwannomas, which is likely due to the inclusion of those limited to the IAC. In our series, eight vestibular schwannomas, which were all related to NF 2 and bilateral, were excluded from this review because of non-surgical therapy during the follow-up period due to lack of symptoms. However, Zuccaro and Sosa noted schwannomas accounted for 24 % among 33 pediatric CPA tumors [7].

According to Phi et al., of 26 children with CPA tumors, 18 (69 %) were malignant and 8 were benign [1]. This contradicts the report by Zuccaro and Sosa indicating benign tumors are more frequent [7]. Of their 27 children with CPA tumors (excluding arachnoid cyst), schwannoma and meningioma are the most common (8 and 6, respectively), 21 (78 %) were benign histologically [7]. In our series, there were 22 malignant tumors (14 ependymomas, 4 ATRTs, 3 PNETs, and 1 glioblastoma) and 22 benign tumors (11 pilocytic astrocytomas, 4 epidermoids, 3 meningiomas, 3 nerve sheath tumors, and 1 ganglioglioma), thus malignant vs. benign tumor ratio being 1:1.

Posterior fossa ependymoma is the most common histology. They originate from ependymal rests of the fourth ventricle. Some take their origin within the lateral recess of the fourth ventricle and grow both laterally into the CPA and medially into the fourth ventricle [9]. When the origin of the tumor is further laterally near the foramen of Luschka, the tumor primarily extends to the CPA and CMF, displacing the brain stem and fourth ventricle contralaterally. In our series, pure CPA localization occurred in only 4 of 14 ependymomas and others had extension to the fourth ventricle as well. These CPA ependymomas rarely invade the floor of the fourth ventricle [10, 11]. Phi et al. reported only 5 of 35 posterior fossa ependymomas in children involving the CPA [1].

JPAs originate often from cerebellar peduncle or lateral brain stem into the CPA/CMF. It may be difficult to distinguish the tumor origin between the brainstem and cerebellar peduncle due to the lack of anatomical borders between them [12]. The patients with exophytic brain stem tumors tend to present with more neurological symptoms than exophytic peduncle tumors. Ganglioglioma similar to one of our cases has been reported in the literature [13].

According to Rorke and Biegel, 52 % of ATRTs occur in the posterior fossa and they stated that the tendency to arise in the CPA with invasion of the surrounding structures is a distinct feature of this tumor [14]. The cell origin in ATRT remains an enigma, but based upon our observation, ATRTs in the CPA appear to originate in the anterolateral cerebellar hemisphere with exophytic extension, displacing both the brainstem and IV ventricle contralaterally. An extremely rare occurrence of ATRT from the acoustic nerve in a young adult was reported [15]. ATRTs usually affect very young children and in this series, all patients were 2 years old or younger, with a mean age of 10 months.

Vestibular schwannomas are very prevalent in adult CPA tumors. However, some report higher incidence of vestibular schwannomas in pediatric CPA; 60 % of pediatric CPA and IAC tumors according to Holman et al. [8], and 86 % of 115 pediatric lateral skull base lesions reported by Cunningham et al. [16]. In most reported series [1, 6, 7], schwannomas are less common than other tumor types in CPA tumors, as in this series. The discrepancy among the series may, in large, part reflect different inclusion criteria for CPA tumors. Pediatric vestibular schwannomas tend to be associated with NF 2, and to affect bilaterally [8]. Due to early screening, vestibular schwannomas of children with NF2 are often smaller and present with a lower incidence of hearing loss than those of sporadic schwannoma cases [8].

CPA epidermoid tumors are considered to be third most common after vestibular schwannoma and meningioma in adults. Of four epidermoids in this series, three cysts contained thick viscid contents whereas another had a typical pearly whitish appearance.

### Presenting symptoms

Tumors of the CPA and CMF often displace the brainstem and cerebellar hemisphere and may cause hydrocephalus. According to Zuccaro and Sosa, about 33 % of patients presented with hydrocephalus [7]. In our series, 23/44 (52 %) presented with hydrocephalus, which was the major cause for presenting symptoms, such as headaches, emesis, lethargy, and gait ataxia and, for infants, macrocephaly and developmental arrest. Localized symptoms include cerebellar dysfunction and cranial nerves paresis, including hearing loss, stridor, and facial weakness. Siu et al. suggested that in children with acute facial palsy due to a CPA tumor, ATRT is highly suspicious [17]. Pre-operative vocal cord status needs to be evaluated by an otolaryngology specialist and speech therapist for post-operative management. In other series, hearing loss was the most common localizing signs [8].

### Surgical approach

A retro-sigmoid approach has been practiced most commonly for the CPA tumors [7]. This approach provides sufficient exposure of the CPA structures but does not allow sufficient exposure of the IV ventricle and structures below the foramen magnum. The retro-sigmoid approach was used in only two of our patients with small CPA tumors. Otherwise, we approached the lesions of CPA/CMF with the patient in prone position through a hockey stick incision, which allows both midline and ipsilateral PF exposure. For the hockey stick incision, we made a midline incision from C2 level to above the inion, then a perpendicular ipsilateral sideward incision above the superior occipital line (above the attachment of suboccipital muscles) to the base of mastoid process (Fig. 1). This approach allows a craniotomy extending far laterally to the ipsilateral mastoid process, medially to the contralateral occipital bone and inferiorly to the upper cervical space. When the tumor is present in the fourth ventricle, as seen more often among ependymomas, the removal of the fourth ventricle portion of the tumor provides cerebellar relaxation, and the entry to the CPA is less laborious because of relaxed cerebellum. Furthermore, the cisterna magna and upper cervical subarachnoid space can be accessed to gain CSF drainage and cerebellar relaxation. Identification of the cervical cord, medulla oblongata and cerebellar vallecula also provide the surgeon with better anatomical orientations. Tracing the spinal accessory nerve rostrally leads to the jugular foramen where the glossopharyngeal and vagal nerves are identified. Sanford et al. describe a lazy “S” incision in which a skin incision is marked from the C2 vertebra up the suboccipital midline and then curved toward behind the ipsilateral ear [10] and this is an alternative to the hockey stick incision we prefer.

### Complications

Although Phi et al. did not disclose the complication details in their report, they state that complication rates for malignant CPA tumors were much higher than the same tumors located in the midline location [1]. Malignant CPA tumors frequently lack arachnoid planes between the tumor and neural structures, encase major arteries and brainstem perforators, and adhere to cranial nerves and brainstem. They had impairment of cranial nerve IX and X and placement of percutaneous gastrostomy occurred only in patients with malignant tumors [1]. Sanford et al. [10] classified surgical complications among children who had resection of CPA ependymomas into minor and major. Minor complications include hoarseness, temporary dysphagia, mild facial weakness, and shunt requirement while major complications are hemiparesis, meningitis, cranial nerve deficits, and vocal cord and swallowing dysfunctions. In their series, there were 14 (31 %) minor and 13 (29 %) major complications including tracheotomy in seven and G-tube in nine patients. Like our patients, however, all but one was decannulated within 1 year of surgery [10]. In other series by Zuccaro and Sosa, among long-term survivors, 10 had no sequelae, 10 mild deficits, and 3 had severe deficits [7].

Injury to the spinal trigeminal tract during the resection of the tumor from the lateral medulla oblongata is likely the cause of trigeminal trophic syndrome occurred in two of patients. Its occurrence in the pediatric population is rare [18].

## Conclusion

The review of our personal series of surgically treated pediatric CPA/CMF tumors shows a plethora of histology types; 50 % are malignant including ependymoma, ATRT, PNET, and glioblastoma, whereas another 50 % were benign consisting of JPA, epidermoid, schwannoma, and meningioma. Common adult tumors seen in adulthood such as schwannoma and meningiomas are uncommon. The caveat of this finding, however, is that this series only included the senior author’s surgical cases. None of intra-canalicular or bilateral vestibular schwannomas was included in this study as they were treated conservatively.

Resecting CPA/CMF tumors remains difficult in children. One should choose appropriate surgical approaches for maximum exposure of the posterior fossa structures and minimum brain retraction. A hockey stick scalp incision with a bilateral posterior fossa craniotomy going far lateral on one side provides wide access to the CPA/CMF and cervical subarachnoid space. However, surgical resections carry high rates of surgical complications, particularly involving the lower cranial nerve deficits. Most of the new neurologic deficits caused from surgery resolves or compensates over time. None-the-less, in order to minimize complications, one should pay meticulous attention during tumor resection from the CPA/CMF. The use of intraoperative neurophysiological monitoring and the surgical microscope is important in order to reduce these complications.
